# Targeting soluble epoxide hydrolase promotes osteogenic–angiogenic coupling via activating SLIT3/HIF‐1α signalling pathway

**DOI:** 10.1111/cpr.13403

**Published:** 2023-01-13

**Authors:** Lu Gao, Weixian Chen, Lijun Li, Juanjuan Li, Wenyao Kongling, Yaoyang Zhang, Xueping Yang, Yanrong Zhao, Jie Bai, Fu Wang

**Affiliations:** ^1^ School of Stomatology Dalian Medical University Dalian China; ^2^ Academician Laboratory of Immune and Oral Development & Regeneration Dalian Medical University Dalian China; ^3^ The Affiliated Stomatological Hospital of Dalian Medical University School of Stomatology Dalian China

## Abstract

Type H vessels have recently been identified to modulate osteogenesis. Epoxyeicostrioleic acids (EETs) have an essential contribution to vascular homeostasis. However, whether increased EETs with soluble epoxide hydrolase (sEH) inhibitor TPPU enhance the coupling of angiogenesis and osteogenesis remains largely unknown. The effects of TPPU on cross‐talk between co‐cultured human umbilical vein endothelial cells (HUVECs) and human dental pulp stem cells (hDPSCs), and on long bone growth and calvarial defect repair in mice were investigated in vitro and in vivo. TPPU enhanced osteogenic differentiation of co‐cultured HUVECs and hDPSCs in vitro and increased type H vessels, and long bone growth and bone repair of calvarial defect. Mechanistically, TPPU promoted cell proliferation and angiogenesis, reclined cell apoptosis, and significantly increased CD31^hi^EMCN^hi^ endothelial cells (ECs) and SLIT3 and HIF‐1α expression levels in co‐cultured HUVECs and hDPSCs. Knockdown of *Slit3* in hDPSCs or *Hif‐1α* in HUVECs impaired the formation of CD31^hi^EMCN^hi^ ECs and reversed TPPU‐induced osteogenesis. We defined a previously unidentified effect of TPPU coupling angiogenesis and osteogenesis. TPPU induced type H vessels by upregulating the expression of hDPSCs‐derived SLIT3, which resulted in the activation of ROBO1/YAP1/HIF‐1α signalling pathway in ECs. Targeting metabolic pathways of EETs represents a new strategy to couple osteogenesis and angiogenesis, sEH is a promising therapeutic target for bone regeneration and repair.

## Graphical abstract

TPPU increases endogenous epoxyeicostrioleic acids (EETs) by inhibiting soluble epoxide hydrolase (sEH). Then, the increased EETs upregulate the expression of SLIT3 in mesenchymal stem cells (MSCs), which results in the activation of ROBO1/YAP1/HIF‐1α signalling pathway in endothelial cells (ECs) and induces type H vessels with high specific expression of CD31 and endomucin (EMCN). Type H vessels promote the cross‐talk between ECs and MSCs and modulate osteogenesis. Thus, targeting metabolic pathways of EETs represents a new strategy to couple osteogenesis and angiogenesis, sEH is a potential therapeutic target for bone repair and regeneration.

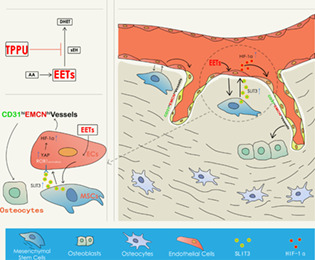

## 1 INTRODUCTION

Bone repair and regeneration of bone defects due to trauma, tumour, infection and skeletal abnormalities are one of the main topics of concern in regenerative medicine, which is a complicated, well‐orchestrated biological process of bone formation.^1^, ^2^ Blood vessels are a prerequisite for bone formation, and serve as a structural template to provide the key signals for bone homeostasis into the osteogenic microenvironment.^3^ Blood vessels can both supply the metabolic substance of bones for nutrients, oxygen and growth factors and provide a way to recruit inflammatory cells, fibroblasts and pre‐osteoblasts/osteoclasts to the injured site.^4^

The cross‐talk between the endothelial cells (ECs) and surrounding cells maintains the molecular microenvironment for osteogenesis. Type H vessels (CD31^hi^EMCN^hi^) have recently been identified to modulate osteogenesis.^5^ They are mainly distributed near the epiphysis in the long bone and are densely surrounded by Runx2 (+) and Osterix (+) bone progenitor cells, which mediate the growth of the vascular system and provide pivotal signals for bone progenitor cells.^6^, ^7^ On the other hand, osteoclasts, osteoblasts and chondrocytes can secrete related factors to regulate EC proliferation and stability.^8^, ^9^ Several molecules have been found to mediate the development of type H vessels, which participate in the coupling of angiogenesis and osteogenesis, including HIF‐1α, Notch, VEGF, and slit guidance ligand 3 (SLIT3).^5^, ^10^, ^11^ Schnurri 3 (SHN3) can impair bone formation by inhibiting extracellular signal‐regulated kinase (ERK) to reduce SLIT3 expression in osteoblasts and type H vessels, providing a possibility for activating the ERK‐SLIT3 pathway of osteoblasts to promote bone formation.^9^

Epoxyeicostrioleic acids (EETs), metabolites of arachidonic acid (AA),^12^ have multiple effects, such as reducing inflammation, promoting angiogenesis, inhibiting osteoclast and adipogenic differentiation of stem cells.^13^, ^14^, ^15^ Especially in the cardiovascular system, EETs have been shown to promote ECs proliferation and angiogenesis in various models.^16^, ^17^ Recent in vivo studies demonstrate that EETs significantly promote liver regeneration, compensatory growth of renal and lung, corneal neovascularization and retinal angiogenesis.^17^, ^18^, ^19^ Our previous research revealed that EETs enhance the repair ability of irradiation‐damaged salivary glands by improving angiogenesis and inhibiting cell apoptosis.^20^ EETs can increase ERK phosphorylation levels and regulate EC function, such as proliferation and migration, through mitotic signalling cascade.^21^ 11, 12‐EET can induce more robust tube formation by markedly increasing VEGF‐A and bFGF expression in myocardial infarction.^22^ EETs also encourage the generation of angiogenesis transcription factors through the HIF‐1α pathway, and increase HIF‐1α‐DNA‐binding activity to improve the resistance of myocytes to acute ischemia–reperfusion.^23^, ^24^, ^25^, ^26^, ^27^

However, soluble epoxide hydrolase (sEH) can rapidly hydrolyze EETs to bio‐inactive dihydroxyeicosatrienoic acids (DHETs) in vivo, so the half‐life of EETs is short, which limits the pharmacological efficacy through EETs administration.^28^ Thus, stabilizing endogenous EETs by sEH inhibitor (sEHi) becomes a candidate strategy. Studies have indicated that sEHi can significantly increase the concentration of EETs in organs, showing the potential of sEHi to be used in humans because it has no obvious toxicity.^29^ TPPU, a potent and highly selective sEHi, shows higher efficiency in inhibiting EETs hydrolysis and stabilizing EETs levels.^30^ Many of the effects of EETs described above are related to angiogenesis, and some of the pathways regulated by EETs also imply that they may be involved in osteogenesis. However, whether increasing EETs levels using TPPU can promote the formation of type H vessels and enhance bone regeneration remains to be determined.

In this study, we define a previously unidentified mechanism by which TPPU links type H vessels and bone formation by targeting sEH. The in vivo results showed that TPPU resulted in high bone mass and CD31^hi^EMCN^hi^ endothelium in mouse femurs and calvarial defect sites. We established an in vitro human umbilical vein endothelial cells (HUVECs) and human dental pulp stem cells (hDPSCs) co‐culture system under osteogenic conditions to study the effect of TPPU on the cross‐talk between them, indicating that TPPU enhanced cell proliferation, angiogenesis and osteogenic differentiation of co‐cultured cells. Mechanistically, TPPU induces a positive feedback loop by upregulating hDPSCs‐derived SLIT3 to stabilize HIF‐1α levels in ECs, thereby promoting the growth of type H vessels to link osteogenesis with angiogenesis. Therefore, our study reveals that TPPU promotes the coupling of osteogenesis and angiogenesis for bone formation by activating SLIT3/HIF‐1α signalling pathway, which provided a novel therapeutic strategy for bone repair and regeneration through targeting sEH.

## 2 MATERIALS AND METHODS

### 2.1 In vivo animal experiments

Experimental animals were provided by the Animal Experiment Center of Dalian Medical University. The animal studies were approved by the Institutional Animal Care and Use Committee of Dalian Medical University (AEE.22004), and obeyed the institution's rules and regulations for using laboratory animals in research. All animal experiments comply with the ethical requirements of ARRIVE.

For femurs bone assay, ten 3‐week‐old mice were divided into two groups randomly and gavaged with or without TPPU (3 mg/kg) every other day. After 2 weeks, the femurs from the euthanized mice were isolated and fixed for 48 h with 4% paraformaldehyde for micro‐CT assay and histochemical staining.

The model of calvarial bone defect as previously described was applied to evaluate the therapeutic benefits of TPPU.^31^, ^32^ Briefly, a critical‐sized (5 mm diameter) circular bone defect was made in the exposed frontal bone of mice under anaesthesia using a microsurgical drill. Plastically compressed collagen gels scaffolds (5 mm diameter) seeded with mice bone marrow mesenchymal stem cells (mBMSCs, 2 × 10^6^ cells/ml) were transplanted into calvarial defect sites.^31^, ^33^ Finally, the skin was sutured, and the animal was observed in accordance with predetermined postoperative guidelines. Skulls were harvested up to 8 weeks after transplantation and were performed micro‐computed tomography (CT) and histological assays.

### 2.2 Micro‐CT analysis

The mouse femurs and skulls were isolated and preserved for 48 h in 4% paraformaldehyde, followed by high‐resolution micro‐CT scanning system (Skyscan 1276, Bruker Micro‐CT). The settings are as follows: 6.5 μm voxels, medium resolution, 85 kV, 200 μA, 1 mm Al filter and 384 ms for integration. The calcium hydroxyapatite phantom was used to assess density. Reconstruction was accomplished by Recon (version 1.7.4.2) and 3D images were contoured from 2D images (CTvox; version 3.3.0). 3D and 2D images were, respectively, analysed by Software CT Analyser (version 1.18.8.0) and ImageJ (Fiji).

### 2.3 Histochemical stain

As directed by the manufacturer, haematoxylin and eosin (H&E) and Masson's trichrome staining were carried out. The immunohistochemistry staining was completed using Histostain‐Plus kits (SP‐9000, ZSGB‐BIO). Briefly, decalcified tissues were deparaffinized with xylene, and then inactivated endogenous peroxidase using 3% H_2_O_2_. After blocking with goat serum (Solarbio) for an hour, the primary antibodies, including anti‐HIF‐1α (Abcam, ab2185, 1:200), anti‐SLIT3 (Santa Cruz Biotechnology, sc‐293463, 1:50) were added to the sections overnight. And then, sections were applied with secondary antibodies and stained with a DAB kit (ZSGB‐BIO). For immunofluorescence, sections were exposed to the primary antibodies (anti‐CD31, ab28364, 1:10; anti‐EMCN, ab106100, 1:200) overnight at 4°C. Then the tissues were incubated with secondary antibodies conjugated with Cy3 568 (ab97075, 1:200) and Dylight 488 (ab96899, 1:200) for 1 h at room temperature. Finally, 4′,6‐diamidine‐2′‐phenylindole dihydrochloride was used to counterstain the nuclei (DAPI, Solarbio). Fluorescent microscopy was used to capture the images (Olympus Corporation) that were analysed as previously mentioned.^34^ All antibodies were from Abcam unless stated.

### 2.4 Cell isolation and culture

This study for isolation and culture of hDPSCs was approved by the Ethics Committee of the Affiliated Stomatological Hospital of Dalian Medical University School of Stomatology per the Declaration of Helsinki (No. 2022001). Premolars extracted for orthodontic treatment were collected from children (14–19 years old) who received orthodontic treatment and all donors provided informed consent in compliance with the applicable rules and regulations. DMEM/F12 (HyClone; 10% FBS) were used for hDPSCs culture following the established method.^34^ In this work, hDPSCs up to passage 5 were used.

The HUVECs (ATCC) were cultured in ECs medium (ECM, ScienCell) supplemented with 5% FBS.

For co‐culture, the HUVECs and hDPSCs were mixed in a 1:1 ratio and then inoculated into the well plates to construct a co‐culture system with direct cell–cell contact between HUVECs and hDPSCs.

Male 5‐week‐old mice's femurs were used to extract mBMSCs, which were then cultivated in α‐MEM medium (HyClone) containing 15% FBS as previously described.^34^ For the in vivo investigation, passages 3–5 of mBMSCs were used.

### 2.5 Cell viability assay

The cells were seeded into a 96‐well plate (1 × 10^3^ cells/well) and treated with or without TPPU (10 μM) for 0, 1, 3, 5 and 7 days. Cell Counting Kit‐8 (CCK‐8; Vazyme Biotech) was used following the manufacturer's protocol. Finally, a microplate reader assessed the optical density at 490 nm (Molecular Devices).

### 2.6 EdU staining assay

The cells were plated (1 × 10^6^ cells/ml) and incubated with EdU (EdU test kit Abcam) for 4 h. The cells were then permeabilized using permeabilization buffer, fixed with 4% paraformaldehyde, and treated with EdU Reaction Mix. Finally, a flow cytometer (BD Pharmingen) was applied to determine how many cells had incorporated EdU at Ex/Em = 491/520 nm.

### 2.7 TUNEL staining

The cells seeded in Coverslips were treated with permeabilization buffer (0.25% Triton‐X‐100) for 5 min. Deparaffinization and permeabilization of the tissue were performed using 20 g/ml Proteinase K in PBS. The apoptotic cells were treated with a TUNEL assay kit (Apoptosis Detection Kit, Vazyme) complied with the manufacturer's instructions and our earlier study.^20^ Finally, DAPI (Sigma‐Aldrich) was used to mount the sections before fluorescence microscopy imaging (Olympus Corporation).

### 2.8 Angiogenesis assay on Matrigel

The tube formation experiment was performed as reported before.^34^ The cells were inoculated in 96‐well plates (1 × 10^4^ per well) precoated with Matrigel (BD Biosciences) and cultured in presence or absence of TPPU (10 μM). The capillary‐like structures were examined with a phase microscope.

### 2.9 Wound healing assay

Cells were added into six‐well plates (2 × 10^5^ cells/well), and a linear scratch was made with a tip after the cells reached 90% confluence. Then, the cells were incubated in a serum‐deprived medium with or without TPPU (10 μM). Image‐Pro Plus software was used to calculate cell migration rate (Media Cybernetics).

### 2.10 Osteogenesis differentiation and analysis

The cells (HUVECs, hDPSCs and co‐culture hDPSCs/HUVECs at a ratio of 1:1) were seeded in six‐well plates (3 × 10^5^ cells/well). When the cells reached 80% confluence, the media was transferred to the osteogenic medium that contains 10% FBS, 0.1 μM dexamethasone (Solarbio), 50 μg/ml L‐ascorbic acid and 10 mM β‐glycerophosphate disodium in α‐MEM culture medium with or without TPPU.^34^ Cells were stained with a fast blue solution after being fixed in 10% neutral formalin buffer to detect ALP activity (Sigma‐Aldrich). Following a wash, the samples were treated with an alkaline buffer solution and phosphatase substrate from Sigma‐Aldrich. The ALP activity was read at the absorbance of 405 nm. For Alizarin red S (ARS) quant assay, the cells after ARS staining was destained with cetylpyridinium conchloride (CPC; Sigma‐Aldrich; 10%) and the supernatant was read at an absorbance of 570 nm.

### 2.11 Western blotting

Proteins were extracted and electroblotted as previously described.^34^ The primary antibodies anti‐HIF‐1α (ab2185, 1:1000), anti‐SLIT3 (R&D System, MN, USA, 1:200), anti‐ALP (ab17973, 1:1000), anti‐RUNX2 (ab23981, 1:1000) and anti‐OCN (ab93876, 1:1000) were added to the strip at 4°C overnight after blocked with 5% BSA (ZSGB‐BIO), the secondary horseradish peroxidase antibody (Solarbio) was then applied to the section for an hour. Finally, labelled membranes were identified by ECL kit (Solarbio) and were assessed by Bio‐Rad VersaDoc image system (Bio‐Rad Laboratories); all primary antibodies used in current study were from Abcam unless otherwise specified.

### 2.12 RT‐qPCR

RNAiso Plus (TRIzol; Vazyme Biotech) was used to extract total RNA from samples. HiScript II Q RT Super Mix (Vazyme Biotech) was applied for reverse transcription. Each experiment employed GAPDH as the internal control. Real‐time quantitative PCR was performed using the ChamQ Universal SYBR qPCR Master Mix (Vazyme Biotech) in the Thermal Cycler Dice Real Time System (TP800, Takara). The RT‐qPCR data were evaluated with the 2^−ΔΔCt^ method. In Additional file 1, the primers were listed: Table S1.

### 2.13 Cell transfection

Transfection of cells was done in accordance with the Genechem manual. The cells were added into six‐well plates (approximately 5 × 10^4^ cells per well) until they reached 50% confluence. Using a recombinant lentivirus, the *sh‐Slit3* plasmid was transfected into hDPSCs and the *sh‐Hif‐1α* plasmid into hHUVECs. The cells were cultured in the media with free FBS and screened with the media containing 0.1% puromycin to remove the untransfected cells. HIF‐1α and SLIT3 levels were measured by western blotting (WB) and RT‐qPCR. The siRNA target sequence was shown in Table S2.

### 2.14 Statistical analysis

At least three times each of the tests were repeated. GraphPad Prism 9 was employed for Student–Newman–Keuls tests and one‐way ANOVA. Data were considered statistically significant at *p* < 0.05 and showed the means ± SEM of replicate measurements.

## 3 RESULTS

### 3.1 TPPU enhances the osteogenic differentiation potential and induces CD31^hi^EMCN^hi^ endothelium formation in co‐cultured HUVECs and hDPSCs

**FIGURE 1:**
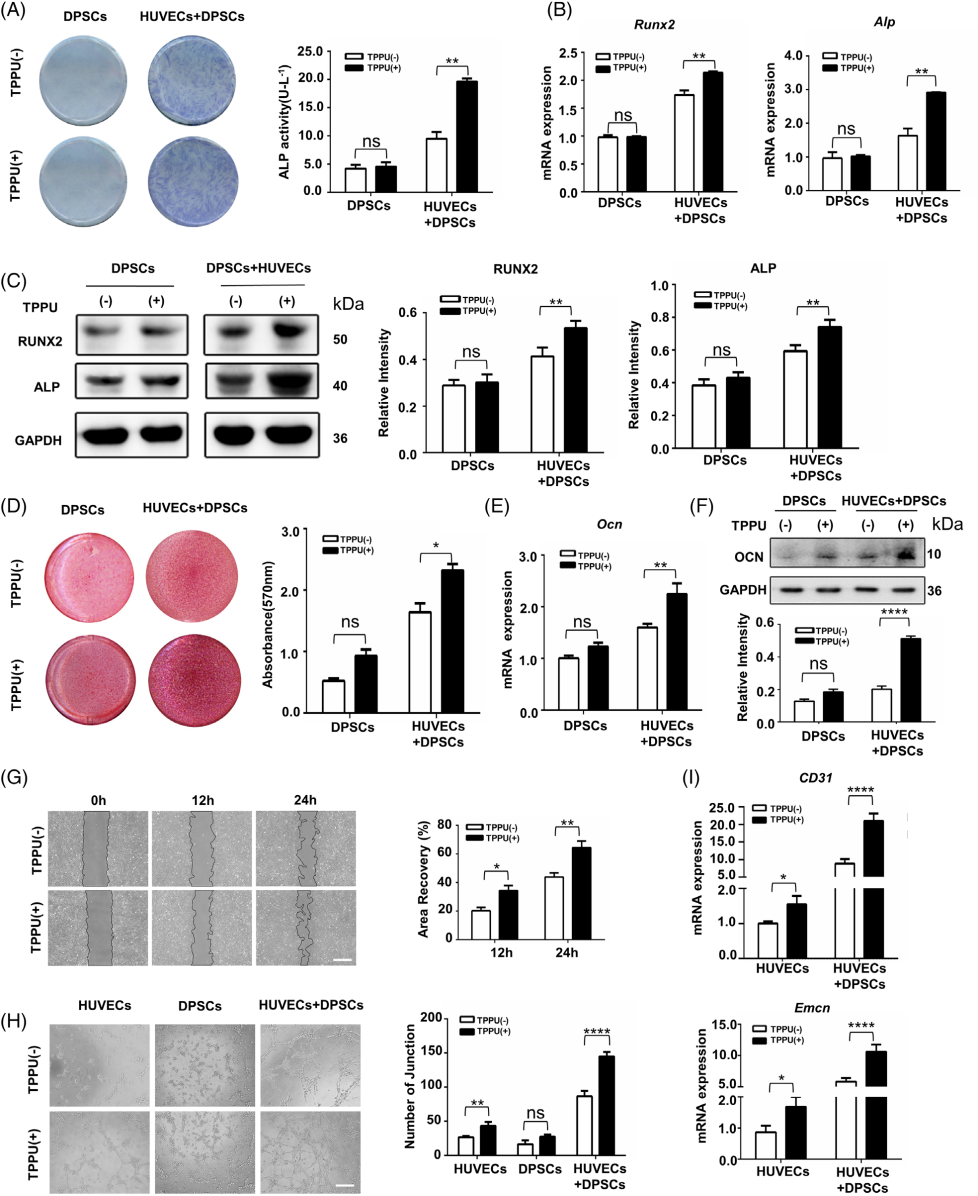
TPPU improves osteogenic differentiation potential and induces CD31^hi^EMCN^hi^ endothelium formation in co‐cultured HUVECs and hDPSCs with direct cell–cell contact. (A) Alkaline phosphatase (ALP) staining and quantitative analysis. (B) RT‐qPCR analysis of *Alp* and *Runx2* expression. (C) Western blotting assay of ALP and Runx2 and quantitative analysis. (D) Representative images of alizarin red staining and relative mineralization assay. (E) RT‐qPCR analysis of *Ocn* expression. (F) Western blotting assay of OCN and quantitative analysis. (G) Representative images and relative quantification of wound healing assays of co‐cultured HUVECs and hDPSCs with or without TPPU treatment (*n* = 3). Scale bar, 200 μm. (H) Representative images and relative quantification of node number of a Matrigel tube formation assay (*n* = 5). Scale bar, 200 μm. (I) RT‐qPCR showing the relative mRNA expression of *CD31* and *Emcn* in co‐cultured HUVECs and hDPSCs after 7 days of osteogenic induction. *p < 0.05; **p < 0.01; ***p < 0.001; ****p < 0.0001. hDPSC, human dental pulp stem cell; HUVEC, human umbilical vein endothelial cell.

TPPU plays considerable effects on angiogenesis and tissue regeneration by specifically inhibiting sEH to increase endogenous EETs. To further investigate the role of TPPU in coupled angiogenesis and osteogenesis, a novel co‐cultured hDPSCs and HUVECs system with direct cell–cell contact was constructed to mimic in vivo cross‐talk between blood vessels and surrounding stem cells. DPSCs are now considered to be a type of mesenchymal stem cells (MSCs) and demonstrate higher clonogenic and proliferative potential than BMSCs. Compared with other MSCs, such as BMSCs, DPSCs are very easily isolated from extracted teeth by low invasive surgery without any ethical issues. Previous studies have confirmed that TPPU can inhibit the adipogenic differentiation of MSCs through increased endogenous EETs, so we first tested the osteogenic differentiation effect of TPPU on hDPSCs and found that TPPU had no obvious promotion for the osteogenesis differentiation of hDPSCs (Figure 1A). Next, we examined the roles of TPPU on osteogenic differentiation of co‐cultured cells. Consistent with previous studies, co‐cultured hDPSCs with HUVECs exhibited higher osteogenic capacity than hDPSCs alone under osteogenic conditions. Interestingly, TPPU further enhanced the osteogenic differentiation ability of co‐cultured HUVECs and hDPSCs. Alkaline phosphatase (ALP) staining showed that TPPU further induced a significantly higher ALP activity in co‐cultured cells. Meanwhile, the *Alp* and *Runx2* expression was markedly upregulated in TPPU‐treated co‐cultured cells as compared to that of vehicle (Figure 1B). WB results also confirmed that the level of ALP and Runx2 proteins was significantly upregulated in TPPU‐treated co‐cultured cells following 7 days of osteogenic induction (Figure 1C). Subsequently, a substantially higher level of mineralization was detected by Alizarin red (AR) staining (Figure 1D) and a strengthen expression of *Ocn* mRNA and protein levels (Figure 1E,F) in co‐cultured cells after 21 days of osteogenic induction. The above results suggested that TPPU did not directly affect the osteogenic differentiation of MSCs alone, implying that TPPU might enhance the osteogenic differentiation of MSCs through acting on ECs to regulate the cross‐talk between HUVECs and hDPSCs.

Because TPPU is known to enhance angiogenesis in vivo and in vitro with upregulating endogenous EETs, we next questioned whether TPPU enhanced the osteogenic differentiation potential of co‐cultured HUVECs and hDPSCs by inducing angiogenesis of type H vessels. To address this, we investigated the role of TPPU on HUVECs alone, hDPSCs alone and co‐cultured HUVECs and hDPSCs, respectively. Scratch assay demonstrated that TPPU promoted moderately enhanced migration ability of HUVECs alone and had no significant effect on cell migration of hDPSCs alone (Figure S1A) and resulted in higher levels of migration in co‐cultured HUVECs and hDPSCs relative to a vehicle control (Figure 1G). TPPU also displayed an enhanced ability to induce tube formation of HUVECs alone, greatly enhance tube formation of co‐culture cells, but had almost no effect on tube formation of hDPSCs alone relative to vehicle control (Figure 1H). Therefore, we suspected that TPPU mainly acted on HUVECs to promote the cross‐talk between HUVECs and hDPSCs, thus increasing the coupling of angiogenesis and osteogenesis in the co‐culture system. Then, we examined the expression of type H vessel‐specific markers CD31 and EMCN. TPPU modestly increased the mRNA level of *CD31* and *Emcn* in HUVECs. Notably, co‐culture of HUVECs and hDPSCs resulted in higher levels of *CD31* and *Emcn* expression, and TPPU treatment further dramatically increased the expression of *CD31* (over 10‐fold) and *Emcn* (over 5‐fold) in the co‐cultured cells relative to vehicle control (Figure 1I). Thus, these results provide proof of the principle that targeting sEH with TPPU contributes to CD31^hi^EMCN^hi^ ECs by enhancing the reciprocal interactions with HUVECs and hDPSCs to induce type H vessel‐related factors.

### 3.2 TPPU increases CD31^hi^EMCN^hi^ ECs via upregulating SLIT3 levels in vitro

**FIGURE 2:**
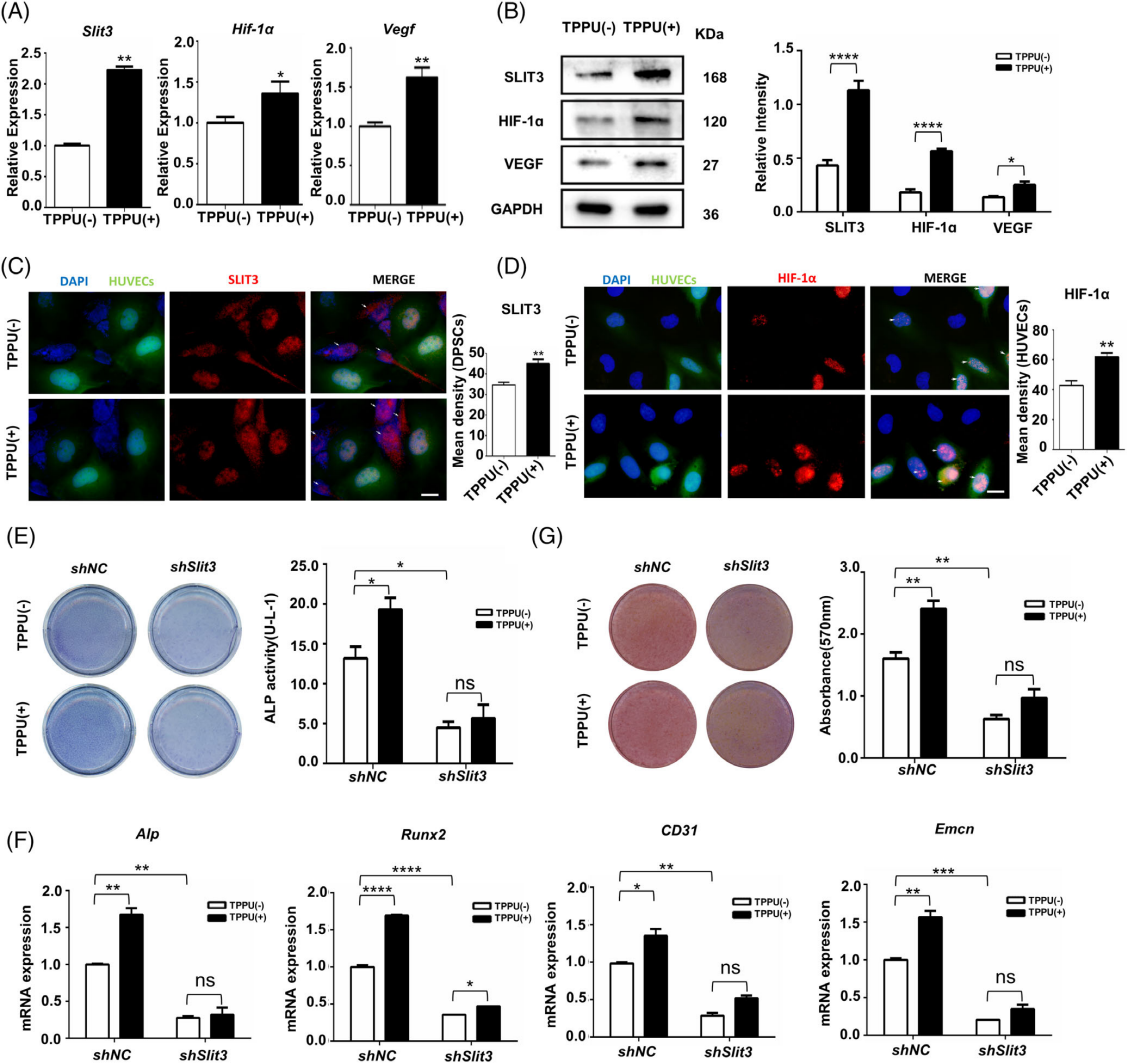
TPPU upregulates SLIT3 levels to promote osteogenesis in vitro. (A) RT‐qPCR showing the relative mRNA expression of *Slit3, Hif‐1α* and *Vegf* in co‐cultured cells. (B) Western blotting assay for protein levels of SLIT3, HIF‐1α and VEGF in co‐cultured cells. (C) Representative images of immunofluorescence staining of co‐cultured HUVECs and hDPSCs after 7 days of osteogenic culture for SLIT3 (red) and HUVECs (green) and quantification of SLIT3 expression. Scale bar, 50 μm. (D) Representative images of immunofluorescence staining of HIF‐1α (red) and HUVECs (green) in the co‐cultured HUVECs and hDPSCs treated with or without TPPU after 7 days of osteogenic culturing and quantitative analysis of HIF‐1α expression. Scale bar, 50 μm. (E) Representative images of ALP staining and ALP activity assay of co‐cultured *Slit3*‐knockdown hDPSCs and HUVECs after 7 days of osteogenesis induction. (F) RT‐qPCR analysis of *Alp*, *Runx2*, *CD31* and *Emcn* in co‐cultured *Slit3*‐knockdown hDPSCs and HUVECs after 7 days of osteogenesis induction. (G) Representative images of alizarin red staining and relative mineralization assay co‐cultured *Slit3*‐knockdown hDPSCs and HUVECs after 21 days of osteogenesis induction. *p < 0.05; **p < 0.01; ***p < 0.001; ****p < 0.0001. hDPSC, human dental pulp stem cell; HUVEC, human umbilical vein endothelial cell.

Given that EETs are known to increase ERK activity, and ERK has been shown to upregulate osteoblast‐derived SLIT3 to increase CD31^hi^EMCN^hi^ endothelium. We hypothesized that TPPU could upregulate osteoblast‐derived SLIT3 to enhance the coupled angiogenesis and osteogenesis. We detected the factors known closely related to type H vessels in co‐cultured HUVECs and hDPSCs. The mRNA levels of *Vegf*, *Hif‐1α* and *Slit3* were greatly increased in co‐cultured cells treated with TPPU as well as protein levels (Figure 2A,B). While TPPU‐treated HUVECs alone only demonstrated a moderate enhancement of both mRNA and protein levels of *Vegf* and *Hif‐1α* (Figure S1B,C). To further identify the cell origin of TPPU‐induced SLIT3 and HIF‐1α, we then detected the role of TPPU on SLIT3 and HIF‐1α expression in the co‐cultured HUVECs (marked with GFP) and hDPSCs under osteogenic induction conditions. In vitro expression analysis in co‐cultured HUVECs and hDPSCs further confirmed that TPPU significantly increased the expression of SLIT3, mainly in hDPSCs (Figure 2C) and induced robust HIF‐1α expression in HUVECs and negligible HIF‐1α expression in hDPSCs (Figure 2D).

To further explore the role of TPPU on SLIT3 coupling angiogenesis and osteogenesis in the co‐culture cells, we performed shRNA‐mediated *Slit*3 knockdown in hDPSCs (Figure S2A). ALP staining showed that *Slit*3 knockdown in hDPSCs significantly impaired the osteogenic differentiation ability of co‐cultured cells, and TPPU could not reverse the impaired osteogenic ability (Figure 2E). Knockdown of *Slit*3 in hDPSCs resulted in decreased mRNA levels of type H vessels‐associated *CD31* and *Emcn*, osteogenic differentiation‐associated *Alp* and *Runx2*, and TPPU could not restore their expression (Figure 2F). Similarly, AR staining showed that knockdown of *Slit*3 in hDPSCs resulted in decreased mineralization activity in co‐cultured cells after 21 days of osteogenic induction (Figure 2G). Therefore, the above results suggested that SLIT3 is indispensable for the osteogenic differentiation of stem cells, and SLIT3 is also necessary for TPPU to participate in osteogenesis and angiogenesis.

### 3.3 TPPU promotes osteogenesis via activating SLIT3‐ROBO1‐YAP1‐HIF‐1α pathway in the co‐cultured HUVECs and hDPSCs

**FIGURE 3:**
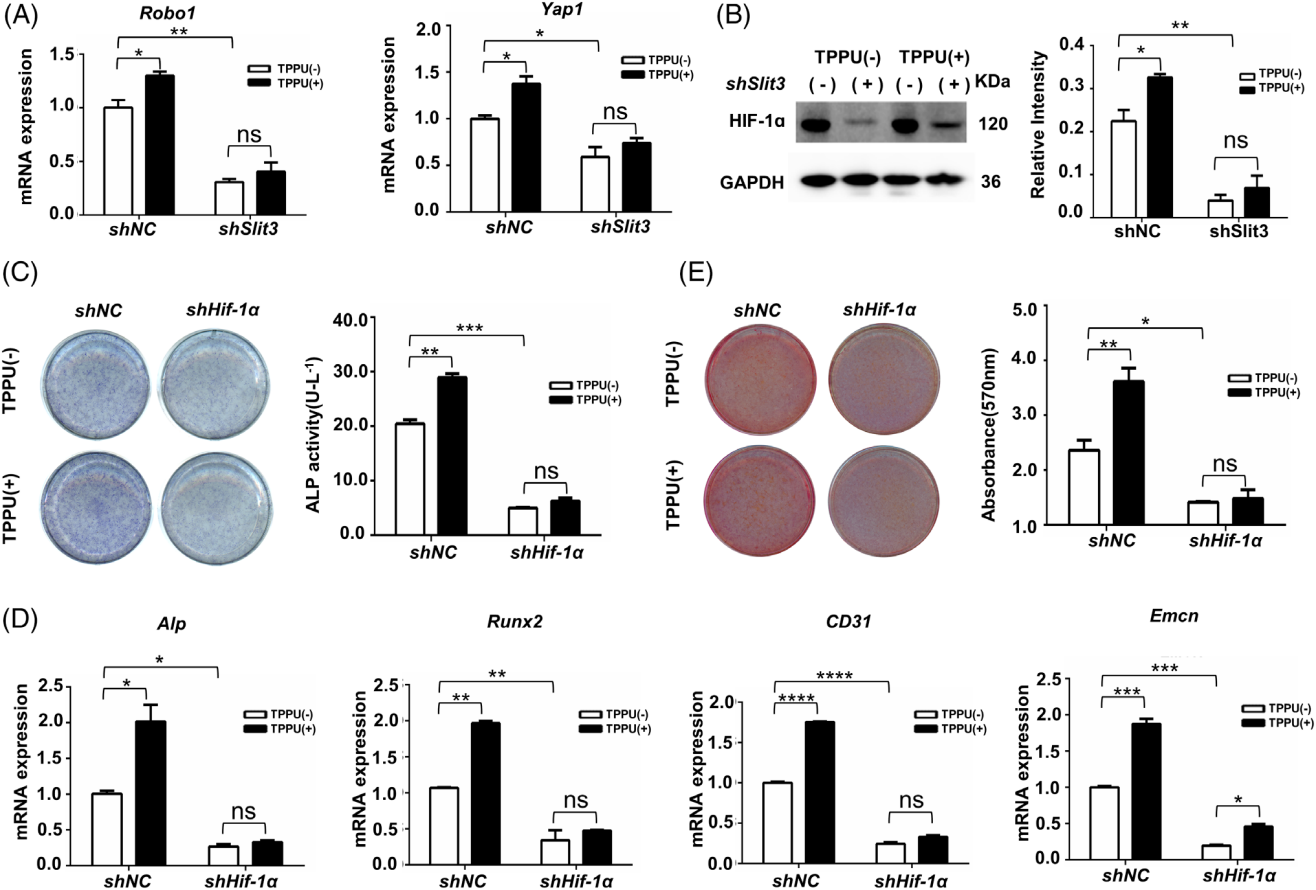
TPPU activates HIF‐1α to enhance type H vessels by increasing osteoblast‐derived SLIT3. (A) RT‐qPCR analysis of *Robo1* and *Yap1* in co‐cultured *Slit3*‐knockdown hDPSCs and HUVECs after 7 days of osteogenesis induction treated with or without TPPU. (B) Western blotting assay and quantification of HIF‐1α expression of co‐cultured *Slit3*‐knockdown hDPSCs and HUVECs after 7 days of osteogenesis induction treated with or without TPPU. (C) Representative images of ALP staining and ALP activity assay of co‐cultured *Hif‐1α* knockdown HUVECs and hDPSCs treated with or without TPPU after 7 days of osteogenesis induction. (D) RT‐qPCR analysis of mRNA expression of *Alp, Runx2*, *CD31* and *Emcn* in co‐cultured *Hif‐1α* knockdown HUVECs and hDPSCs treated with or without TPPUs after 7 days osteogenic induction. (E) Representative images of alizarin red staining and relative mineralization assay of co‐cultured *Hif‐1α* knockdown HUVECs and hDPSCs treated with or without TPPUs after 21 days of osteogenesis induction. *p < 0.05; **p < 0.01; ***p < 0.001; ****p < 0.0001. hDPSC, human dental pulp stem cell; HUVEC, human umbilical vein endothelial cell.

Osteoblasts‐derived SLIT3 mediate communication between osteoblasts and CD31^hi^EMCN^hi^ ECs via activating roundabout guidance receptor 1 (ROBO1)‐Yes‐associated protein 1 (YAP1) pathway in ECs. We then investigated the signalling pathways downstream of SLIT3 to detect the effect of TPPU on cross‐talk between hDPSCs and HUVECs under osteogenic conditions. TPPU increased the expression of *Robo1* and *Yap1* in co‐cultured cells, which was notably decreased after *Slit3* knockdown in hDPSCs, and TPPU could no longer rescue their expression (Figure 3A). The protein levels of HIF‐1α, a well‐known factor for type H vessels, were considerably reduced in the co‐cultured cells following *Slit3* knockdown in hDPSCs, TPPU only led to a negligible enhancement in the expression of HIF‐1α (Figure 3B), demonstrating that TPPU mediated HIF‐1α expression through SLIT3‐ROBO1‐YAP1 axis.

Hypoxia‐regulated factors have profound effects on bone development and remodelling. To further explore the role of TPPU involved in angiogenesis and osteogenic coupling through HIF‐1α signalling pathway, we transduced HUVECs with retrovirus that induced knockdown of *Hif‐1α* by RNA‐mediated interference (Figure S2B), which were co‐cultured with hDPSCs. Notably, knockdown of *Hif‐1α* expression in HUVECs impaired osteogenic differentiation ability of co‐cultured cells by ALP staining and AR staining and abrogated the effect of bone formation enhanced by TPPU (Figure 3C,E). As expected, knockdown of *Hif‐1α* in HUVECs also caused a substantial decrease of type H vascular‐related genes (*CD31* and *Emcn*) and osteogenesis‐related genes (*Runx2* and *Alp*) after 7 days of osteogenic induction, and TPPU did not reverse the expression of these genes (Figure 3D), further confirmed that TPPU mainly induced an increase of HIF‐1α expression in ECs. Moreover, previous studies have proved that HIF‐1α could directly bind to the promoters of SLIT3. However, we did not observe evidence that knockdown of *Hif‐1α* in HUVECs resulted in a dramatic decrease in *Slit3* (Figure S3). Taken together, these results further suggested that TPPU further enhanced HIF‐1α expression in ECs mainly by upregulating hDPSCs‐derived SLIT3, thereby promoting the coupling of osteogenesis and angiogenesis.

### 3.4 TPPU enhances bone formation, bone repair and increases type H vessels in vivo

**FIGURE 4:**
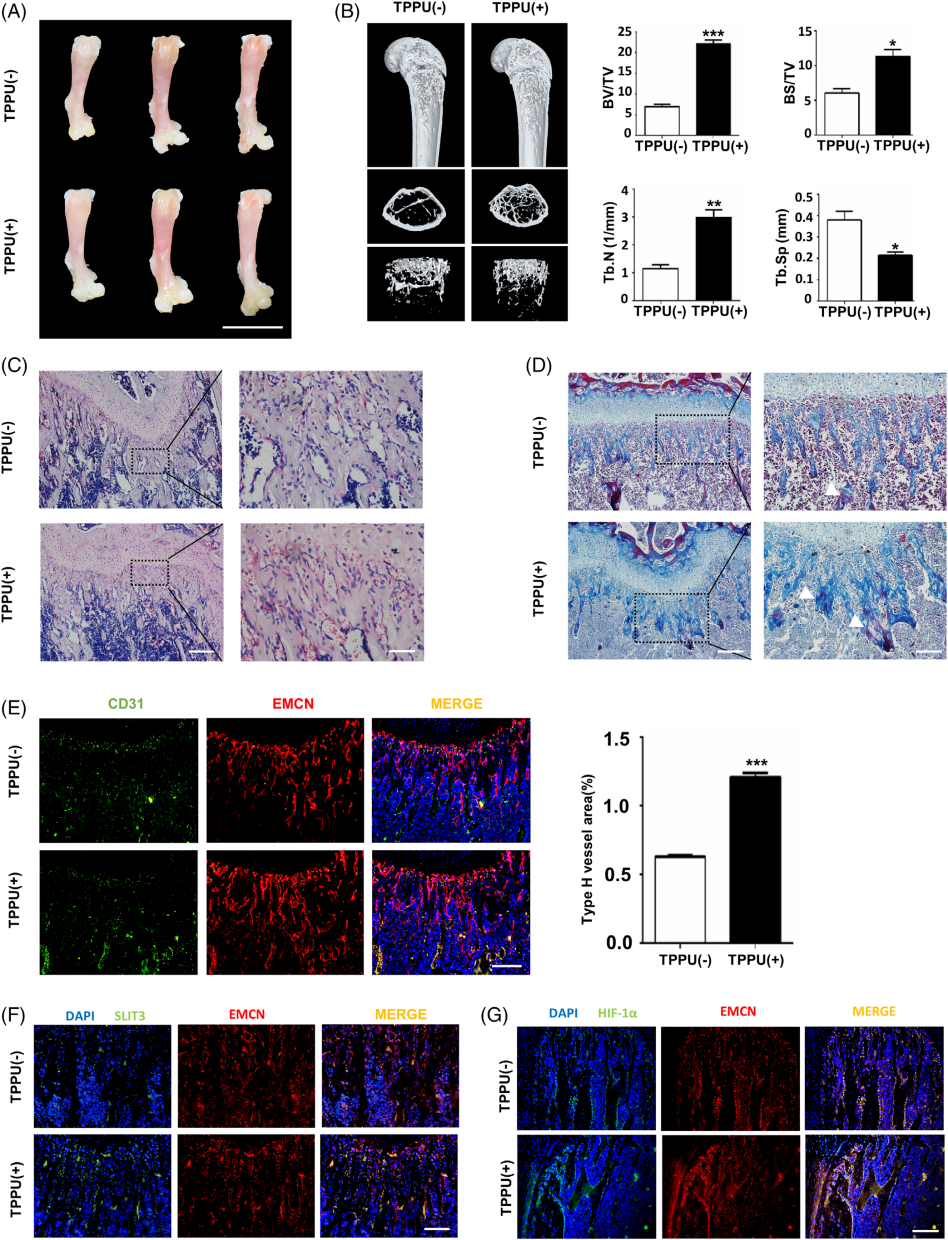
TPPU enhances bone formation in mice. Three‐week‐old C57/BL6 mice were gavaged with TPPU or the same volume of PBS every other day for 2 weeks, and then the femurs were isolated. (A) Representative images of the femurs isolated from mice. Scale bar, 1 cm. (B) Representative micro‐CT images showing 3D‐reconstruction of the femoral metaphysis and quantitative analysis of trabecular bone volume fraction (BV/TV), bone surface per total volume (BS/TV), trabecular number (Tb.N) and trabecular separation (Tb.Sp). (C) Representative HE staining of the femur sections. White arrows indicate blood vessel‐like structures. Magnified areas of dashed boxed sections are shown in right panels. Scale bar, 200 μm (left panel), 50 μm (right panel). (D) Representative Masson's trichrome staining of the femur sections. White arrows represent collagen fibres (blue). Magnified areas of dashed boxed sections are shown in right panels. Scale bar, 200 μm (left panel), 100 μm (right panel). (E) Representative immunofluorescence staining of mouse femur sections for CD31 (green) and EMCN (red) and the quantitative analysis, Scale bar, 100 μm. (F) Representative immunofluorescence staining of mouse femur sections for SLIT3 (green) and EMCN (red), Scale bar, 100 μm. (G) Representative immunofluorescence staining of mouse femur sections for HIF‐1α (green) and EMCN (red), Scale bar, 100 μm. *n* = 4. *p < 0.05; **p < 0.01; ***p < 0.001. CT, computed tomography.

To further investigate whether TPPU contributes to bone growth in vivo, we administered TPPU by oral gavage every other day to three‐week‐old C57BL/6 mice for 2 weeks to determine whether TPPU contributes to bone formation. Then, we examined the long bones and found that the freshly isolated femurs looked brighter red and larger in size in TPPU‐treated mice, implying that TPPU seems to have an effect on angiogenesis and bone growth (Figure 4A). We next evaluated whether TPPU promoting long bone growth was associated with cell proliferation and apoptosis. Cell proliferation is known to be promoted by the co‐culture of MSCs and ECs. CCK8 showed that TPPU further enhanced the proliferation of co‐cultured cells (Figure S4A), and EdU labelling showed a similar increase compared with vehicle (Figure S4B). The apoptotic cells were significantly less in TPPU‐treated co‐cultured cells by TUNEL staining (Figure S4C). Consistent with the in vitro data, in vivo immunohistochemistry (IHC) and IF analysis showed that TPPU significantly increased Ki67‐positive cells (Figure S4D) and decreased TUNEL‐positive cells in the femoral metaphysis of C57/BL6 mice (Figure S4E). Our results demonstrated that TPPU might be involved in bone growth by coordinating cell proliferation and apoptosis.

Micro‐CT analysis further confirmed that TPPU resulted in a higher bone mass and density (Figure 4B). In addition, we also found that increased blood vessels and collagen by HE and Masson's trichrome staining in TPPU‐treated mice femur compared with vehicle mice (Figure 4C,D). EMCN is known to be highly enriched in kidney and lung, and we found that TPPU could promote CD31^hi^EMCN^hi^ ECs in the kidneys and lungs of C57/BL6 (Figure S5A,B). Given that type H vessels modulate bone sculpting and remodelling, we performed immunofluorescence (IF) colocation staining to measure the abundance of CD31^hi^EMCN^hi^ vessels in the epiphysis. Notably, TPPU treatment considerably increased type H vessels with CD31^hi^EMCN^hi^ endothelium beneath the growth plate in the femur (Figure 4E). Consistent with in vitro results, TPPU induced substantially greater levels of SLIT3 and HIF‐1α expression near the growth plate of femur. Immunofluorescence analysis confirmed that most of the SLIT3‐positive cells were adjacent to EMCN‐expressing cells (Figure 4F, a few co‐localized with EMCN), and an increase of HIF‐1α expression co‐localized with EMCN in the femoral metaphysis (Figure 4G).

**FIGURE 5:**
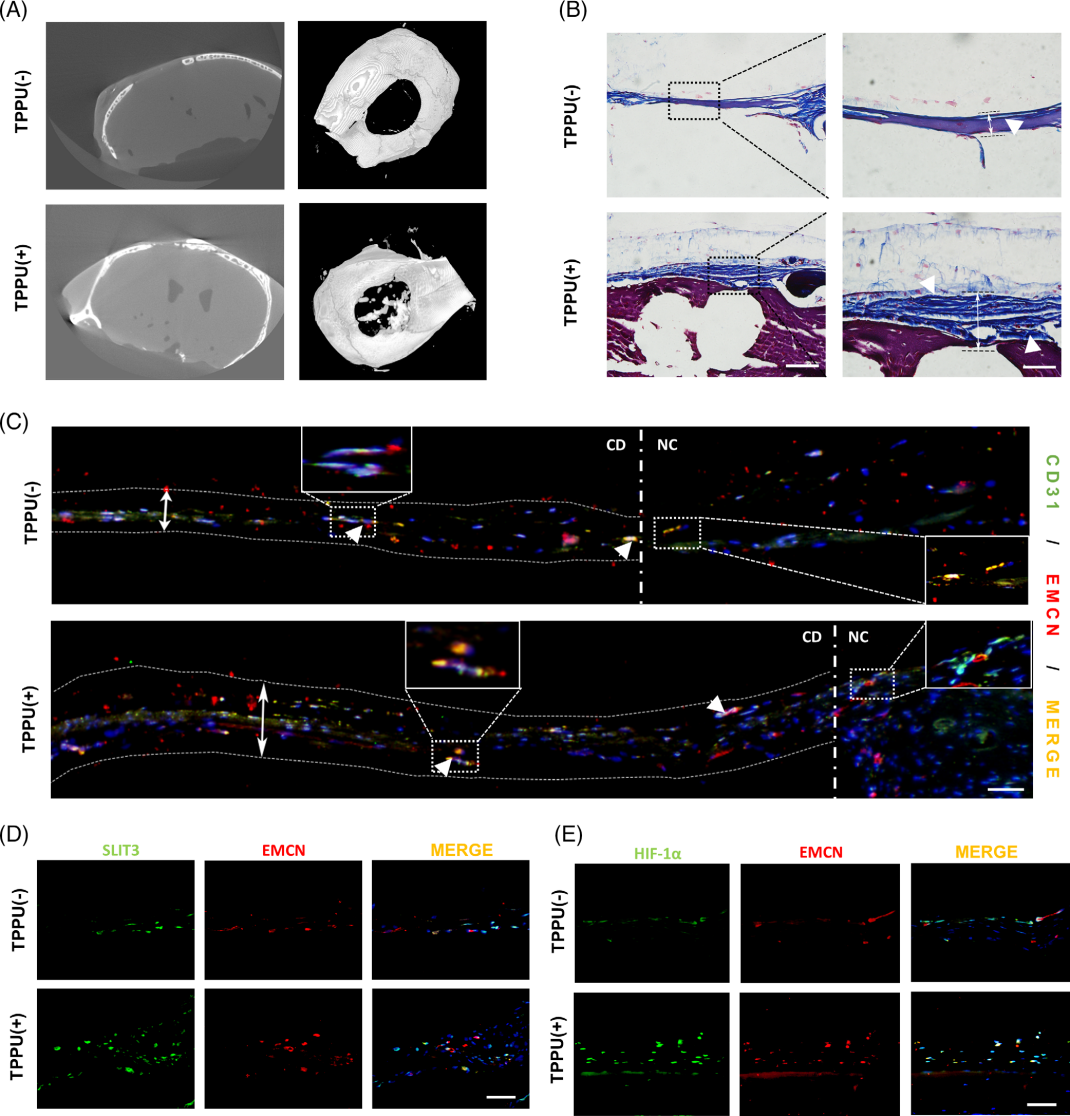
TPPU promotes bone regeneration in the calvarial defect model. A 5‐mm‐size defect was made in the mouse calvaria and mBMSCs were transplanted into the calvarial defect region. The mice were gavaged with or without TPPU treatment. (A) Micro‐CT images for the bone repair in calvarial defects of different groups after 8 weeks of implantation. (B) Representative Masson's trichrome staining of the sections of calvarial defect region. White arrows represent collagen fibres (blue). Magnified areas of dashed boxed sections are shown in right panels. Scale bar, 200 μm (left panel), 100 μm (right panel). (C) Representative immunofluorescence staining of mouse calvarial defect region sections for co‐expressing CD31 (green) and EMCN (red) endothelial cells (arrowhead). Magnified areas of dashed boxed sections are shown. CD, calvarial defect; NC, normal calvaria. Scale bar, 100 μm. (D) Representative immunofluorescence staining of mouse calvarial defect region sections for SLIT3 (green) and EMCN (red), Scale bar, 100 μm. (E) Representative immunofluorescence staining of mouse calvarial defect region sections for HIF‐1α (green) and EMCN (red), Scale bar, 100 μm. *n* = 4. CT, computed tomography; mBMSC, mice bone marrow mesenchymal stem cell.

To evaluate whether TPPU has the same effect on bone repair, the critical‐size calvarial defects in mice were applied to test the therapeutic effects of TPPU on bone regeneration. The bone repair efficiency inside the defect regions 8 weeks after mBMSCs implantation was assessed using radiographic and histological methods. The significantly more regenerated bone tissue in the TPPU‐treated mice than in the control mice, according to the rebuilt morphology of the defect locations (Figure 5A). Masson's trichrome staining in calvarial defects showed thicker regenerated tissue and more collagen fibres in TPPU‐treated mice (Figure 5B). Immunofluorescent staining showed that TPPU increased the number of positive cells with high expression for CD31 and EMCN (Figure 5C), SLIT3 (Figure 5D) and HIF‐1α (Figure 5E) in defect area. Therefore, the findings demonstrated that oral TPPU administration induced type H vessels which enhanced bone growth and bone repair.

## 4 DISCUSSION

The relationship between blood vessels and osteoblasts affects how bone metabolism is balanced. Through certain vascular shapes and routes, angiogenesis and osteogenesis are connected. Blood vessels act as structural models for the creation of new bone and are the primary regulators of bone regeneration.^26^, ^27^ Early revascularization is essential for bone repair and tissue engineering regeneration. Research in recent years has suggested that it is conducive to bone development and stability by promoting or maintaining type H vessels coupling osteogenesis.^35^ Matrix metalloproteinase‐9, HIF‐1α and VEGF released by ECs from type H vessels promote cartilage absorption to enhance longitudinal bone growth.^27^ Our in vivo study demonstrated that increased EETs levels by targeting sEH with TPPU increased type H vessels and enhanced bone formation. The co‐culture experiments in vitro showed that TPPU could significantly enhance the abilities of cell proliferation, tube forming and migration of the co‐culture of vascular ECs and MSCs. Moreover, TPPU might promote osteogenic differentiation via upregulating the expressions of HIF‐1α and SLIT3, the key factors of type H vessels coupling osteogenesis in the co‐cultured cells, and significantly promoted osteogenic differentiation. Further study confirmed that TPPU induced type H vessels by increasing the expression of hDPSCs‐derived SLIT3 which resulted in the activation of ROBO1/YAP1/HIF‐1α signalling pathway in ECs. These results provide evidence that TPPU enhances the coupling of angiogenesis and osteogenesis.

Increasing evidence indicates that EETs, derived from AA metabolized by CYP450 epoxygenase, have an essential role in vascular homeostasis. EETs widely exist and act on ECs and smooth muscle cells, which can affect vascular tension and resistance, and regulate vascular proliferation and growth.^17^, ^18^, ^36^ Chen et al. first found that EETs might affect cell signal transduction and proliferation in renal epithelial cells.^37^ With the gradual deepening of research, the mechanism of EETs promoting angiogenesis has gradually become clear. Angiogenesis is an important link to maintaining the normal vascular network structure and ensuring the nutrition and blood supply of organs and tissues.^38^ High expression of *CYP2C* gene in coronary artery ECs can significantly increase the level of 11,12‐EET and the number of vascular ECs.^39^ Similarly, in cultured pulmonary vascular ECs, 11,12‐EET and 14,15‐EET can encourage the proliferation of vascular ECs by boosting the phosphorylation levels of protein kinase B and ERK.^40^ Webler et al. showed that it could significantly reduce the ability of ECs budding and tubulation induced by VEGF by inhibiting the expression of CYP2C, thereby reducing the level of EETs.^41^, ^42^ Other studies have demonstrated that EETs inhibit osteoclastogenesis through modulation of multiple pathways both upstream and downstream of RANKL signalling.^42^

Kusumbe et al.^5^ found a unique vascular subtype near the growth plate of trabecular bone and cortical bone in mice, as well as on the surface of periosteum and intima. In view of the difference of ECs surface markers, it is classified into H‐type vessels and L‐type vessels. H‐type vessels are located in the periosteum and inner layer of the shaft, under the articular cartilage, and near the growth plate of the metaphysis. A large number of Osterix (+) bone progenitor cells gather around H‐type vessels, which stimulate the growth and differentiation of bone marrow progenitor cells by producing specific factors, and actively guide bone formation.^43^ Many factors such as PDGF‐BB,^8^ Notch signal,^44^ miR497‐195 cluster^45^ and controlled mechanical loading^46^ are identified to regulate H‐type vessels. Recent studies have shown that SLIT3 is a new vascular factor, and deletion of *Slit3* gene reduces CD31^hi^EMCN^hi^ ECs in bone, thereby affecting the formation of new bone after injury.^9^ SLIT3 can regulate osteoclasts and osteoblasts at the same time.^47^ SLIT3 is activated by β‐Catenin, which stimulates osteoblast migration and proliferation, and also inhibits osteoclast differentiation through autocrine, thereby inhibiting bone resorption. Mice that knockdown *Slit3* or its receptor *Robo1* exhibit an osteopenia phenotype as a result of increased bone resorption and reduced bone formation. SLIT3 is mainly expressed in stem cells, but also expressed to a certain extent in ECs and vascular smooth muscle cells in vasculature, suggesting that SLIT3 may act on ECs in paracrine and autocrine manners.^48^ In our study, TPPU significantly increased the expression of SLIT3 in mouse metaphysis. And it is expressed in the same region as EMCN‐labelled ECs, indicating that there may be interaction between these two kinds of cells, and SLIT3 and EMCN play an important role in this process. Our results also suggest that knockdown of *Slit3* in hDPSCs significantly impairs TPPU‐enhanced the coupling of osteogenesis and angiogenesis. However, TPPU has no obvious change on the expression of SLIT3 in ECs, so we did not separately detect the effect of SLIT3 knockdown on angiogenesis in ECs, which will be our further research work.

The ability of bone marrow‐derived ECs to form tubes and the phosphorylation of the hippo pathway signalling intermediary YAP1 show that Robo1 knockdown affects how these cells respond to SLIT3.^9^ According to previous research, YAP1 is essential for ECs migration and tube formation.^49^, ^50^ Our study found that TPPU could upregulate the expression of ROBO1 and YAP1 in the co‐culture cells, but when SLIT3 was inhibited, the effects of TPPU on ROBO1 and YAP1 were blocked. When *Slit3* was knocked down, HIF‐1α upregulated by TPPU was significantly inhibited. HIF‐1α is a heterodimer transcription factor that regulates both normal and abnormal angiogenesis and governs how cells react to oxygen fluctuations.^51^, ^52^ Many studies have proved that HIF‐1α knockdown in ECs could reduce the ability of angiogenesis through a variety of signal pathways. Endothelial HIF‐1α has been shown to be a significant activator of H‐type vessels development in the metaphysis. Osteoprogenitors were significantly decreased after EC‐specific ablation of HIF‐1α, which was followed by a decrease in trabecular bone formation.^5^, ^53^ In the nucleus, YAP1 activation directly interacts with HIF‐1α and maintains HIF‐1α stability to stimulate the transcription of genes associated to angiogenesis.^54^ Our results also confirm that HIF‐1α is mainly induced by the MSC‐derived SLIT3 upregulated by TPPU, while HIF‐1α has little effect on SLIT3. Given that TPPU has little effect on the osteogenic differentiation of hDPSCs alone, suggesting that the cross‐talk between ECs and MSCs plays a critical role, the detailed mechanism of TPPU regulating SLIT3 expression in stem cells through ERK requires further investigation. Furthermore, systemic administration demonstrates the effect of TPPU in coupling angiogenesis and osteogenesis. It is worth looking forward to improving the drug delivery method for local application in the future.^55^, ^56^

Thus, we prove that TPPU promotes type H vessels formation and enhances bone formation, and provides new evidence linking TPPU to the coupling of osteogenesis and angiogenesis via SLIT3‐ROBO1/YAP‐HIF‐1α signalling axis, highlighting that inhibiting sEH to enhance the coupled angiogenesis and osteogenesis can be considered a potential strategy for bone repair and regeneration.

## AUTHOR CONTRIBUTIONS

Fu Wang contributed to the study concept and design. Lu Gao, Weixian Chen and Lijun Li performed qPCR and western blot experiments, performed statistical analysis and designed final figure layout. Juanjuan Li, Wenyao Kongling, Yaoyang Zhang, Xueping Yang and Yanrong Zhao analysed histological data, helped perform immunohistochemistry and assisted with sample harvests. Weixian Chen and Jie Bai performed micro‐CT test and analysis. Fu Wang and Lu Gao analysed the data, drafted and revised the manuscript. All authors reviewed and approved the final manuscript.

## CONFLICT OF INTEREST

The authors declare no conflict of interest.

## Supporting information


**FIGURE S1.** Effects of TPPU on HUVECs and hDPSCs alone migration and vascular‐related gene expression

**FIGURE S2.** Knockdown of *Slit3* and *Hif‐1α*

**FIGURE S3.** HUVECs (*Hif‐1α*‐knockdown) and hDPSCs were co‐cultured with or without TPPU for 7 days

**FIGURE S4.** TPPU promotes cell proliferation and inhibits cell apoptosis

**FIGURE S5.** TPPU treatment increases EMCN expression in lung and kidney

**TABLE S1.** Primer sequence of the target genes
**TABLE S2.** Target sequence of siRNA

## Data Availability

The original data sets in current study are available from the corresponding author on reasonable request.
