# Supplementary figures and images for: Targeting soluble epoxide hydrolase promotes osteogenic–angiogenic coupling via activating SLIT3/HIF‐1α signalling pathway
Source: Cell Prolif. 2023 Jan 13;56(7):e13403. doi: 10.1111/cpr.13403 (PMC10334284; doi:10.1111/cpr.13403)

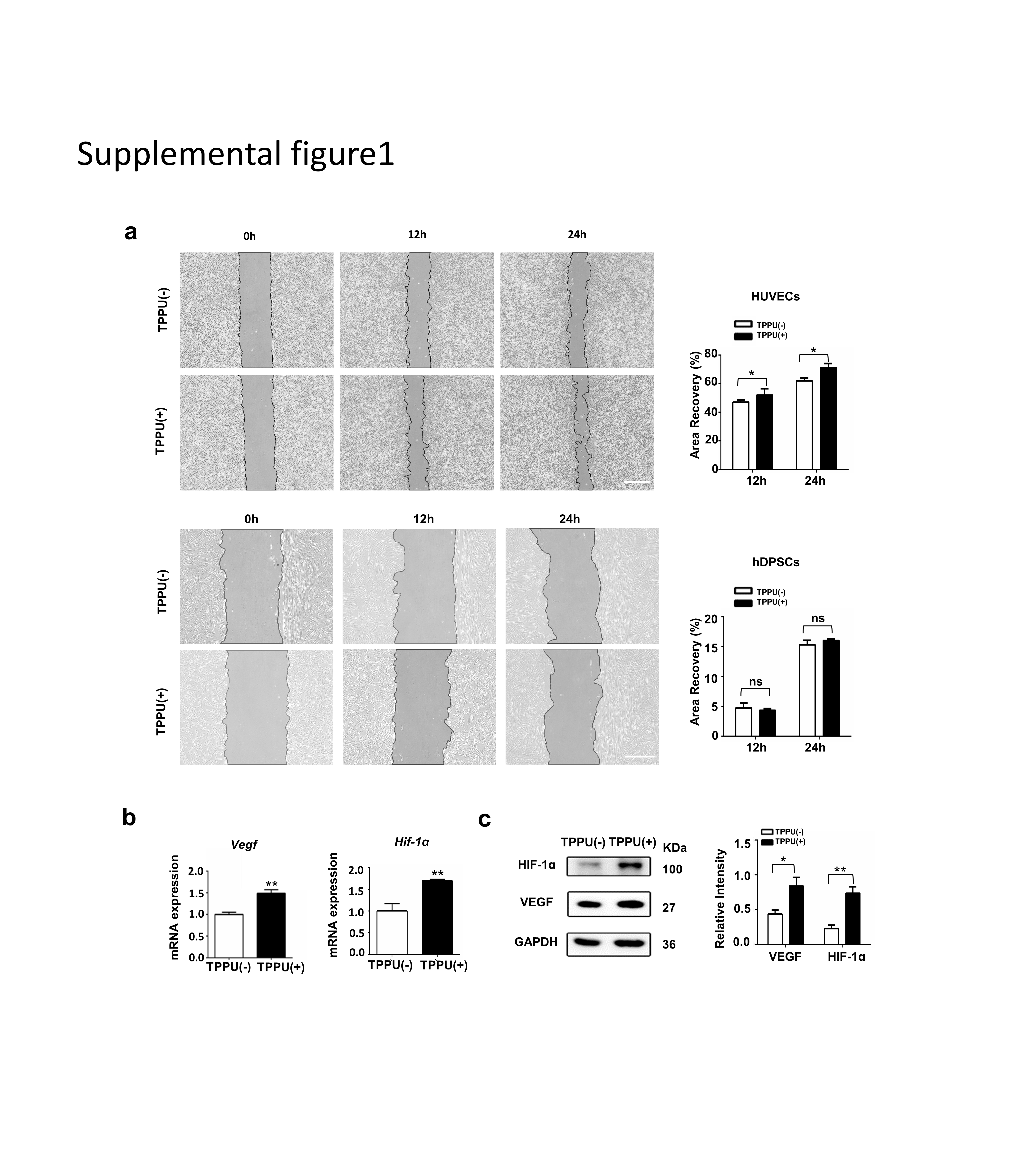

Supplement: Supplementary file 1 — FIGURE S1. Effects of TPPU on HUVECs and hDPSCs alone migration and vascular‐related gene expression [file CPR-56-e13403-s001.TIF]

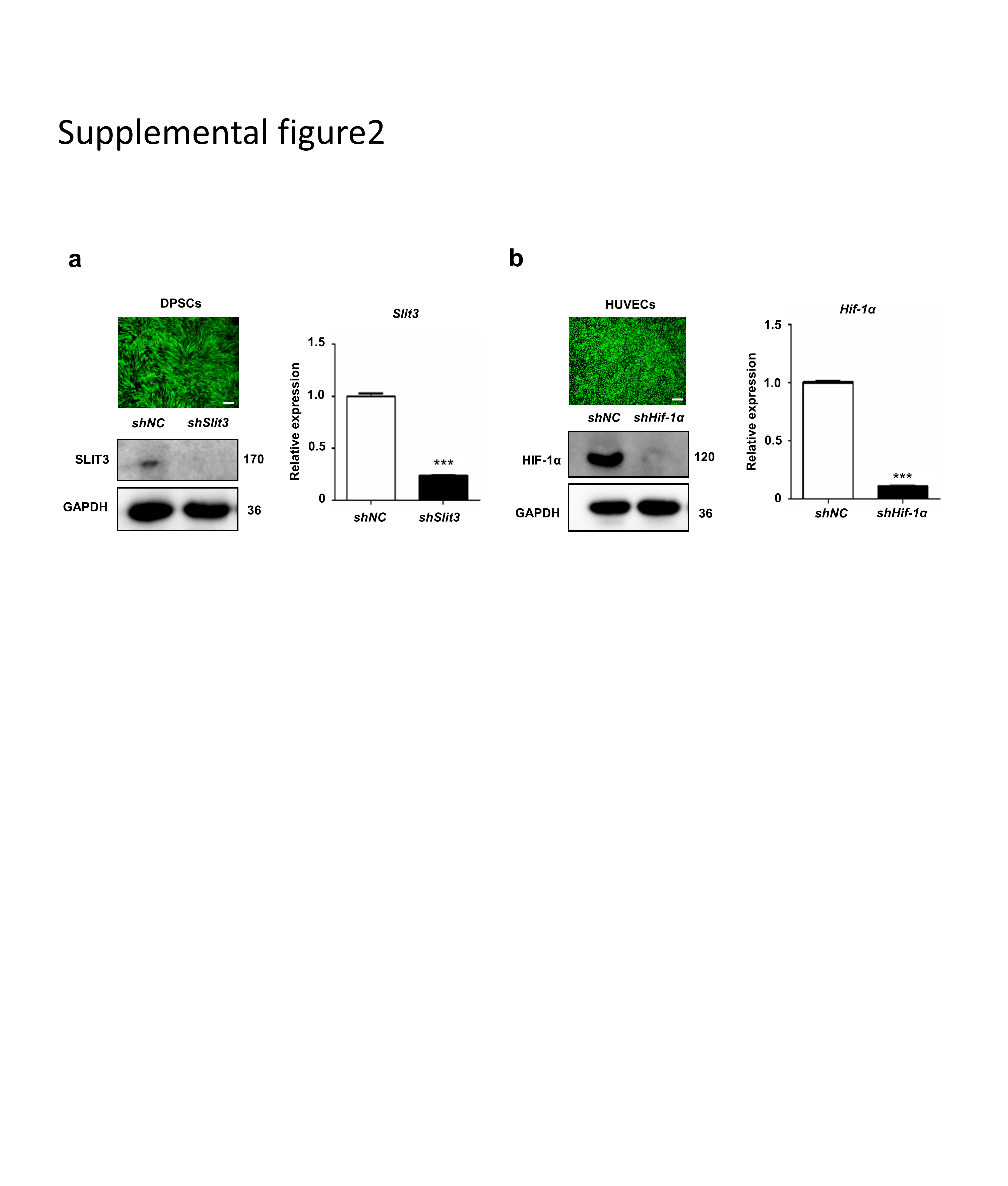

Supplement: Supplementary file 2 — FIGURE S2. Knockdown of Slit3 and Hif‐1α [file CPR-56-e13403-s006.TIF]

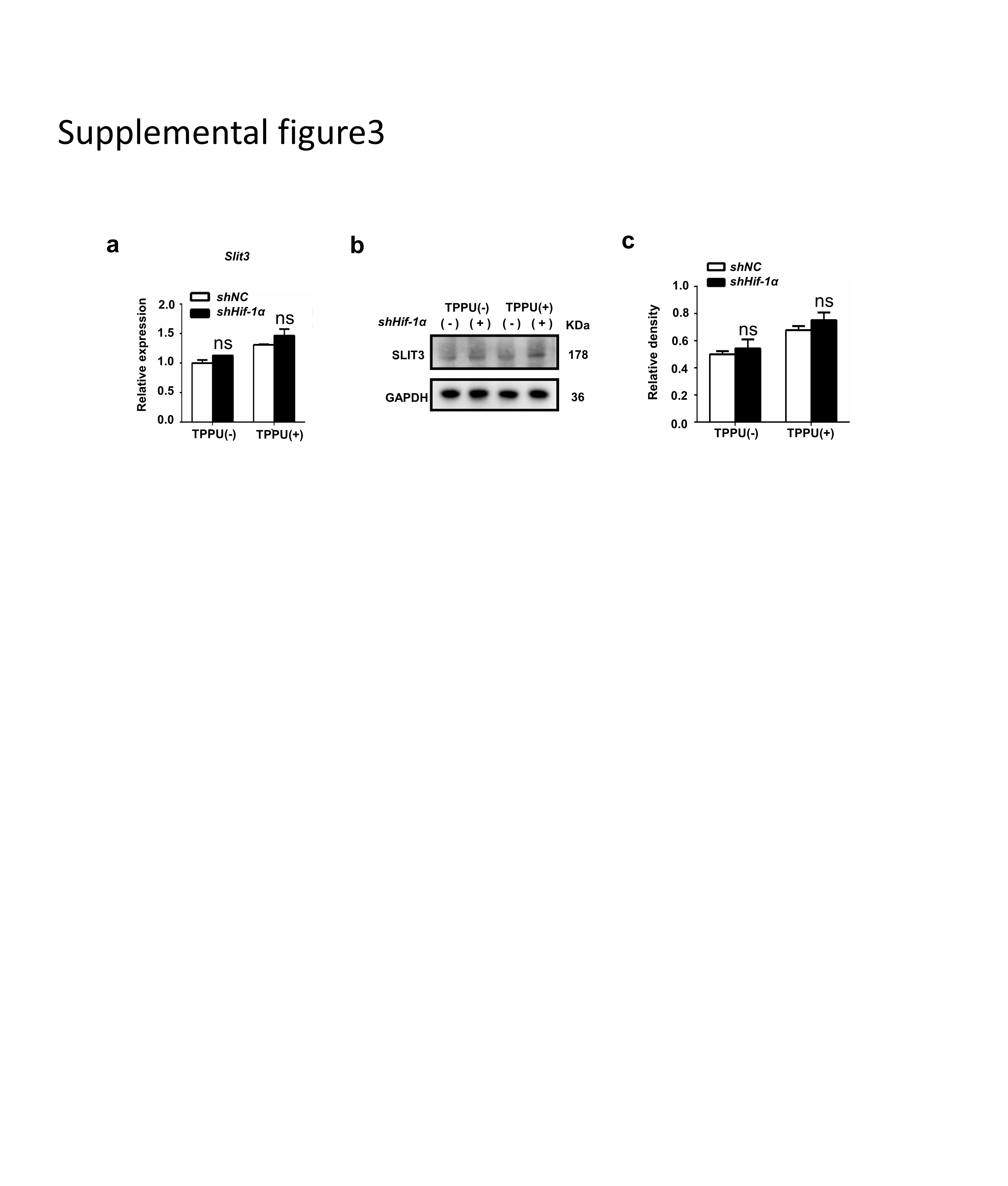

Supplement: Supplementary file 3 — FIGURE S3. HUVECs (Hif‐1α‐knockdown) and hDPSCs were co‐cultured with or without TPPU for 7 days [file CPR-56-e13403-s004.TIF]

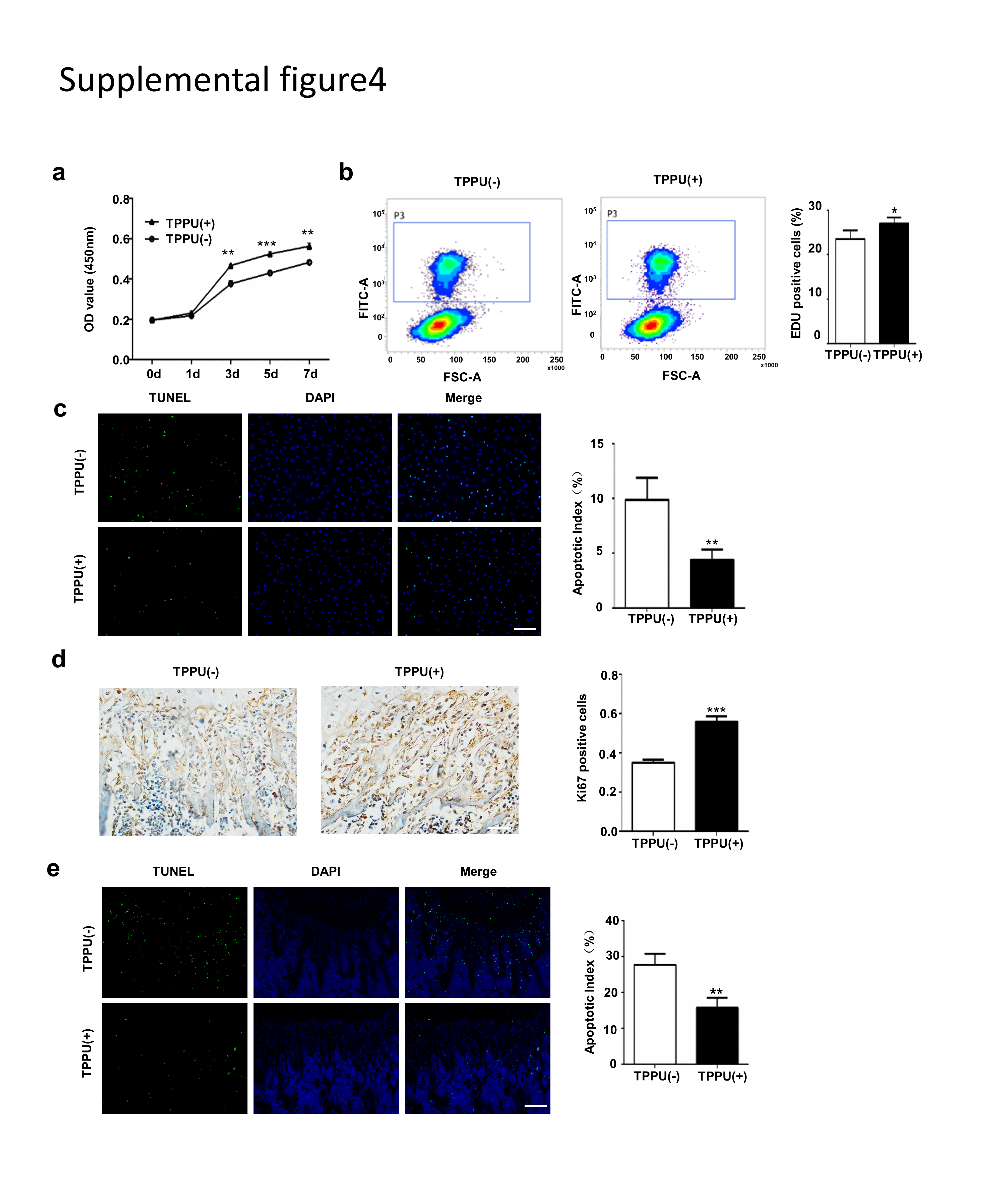

Supplement: Supplementary file 4 — FIGURE S4. TPPU promotes cell proliferation and inhibits cell apoptosis [file CPR-56-e13403-s005.TIF]

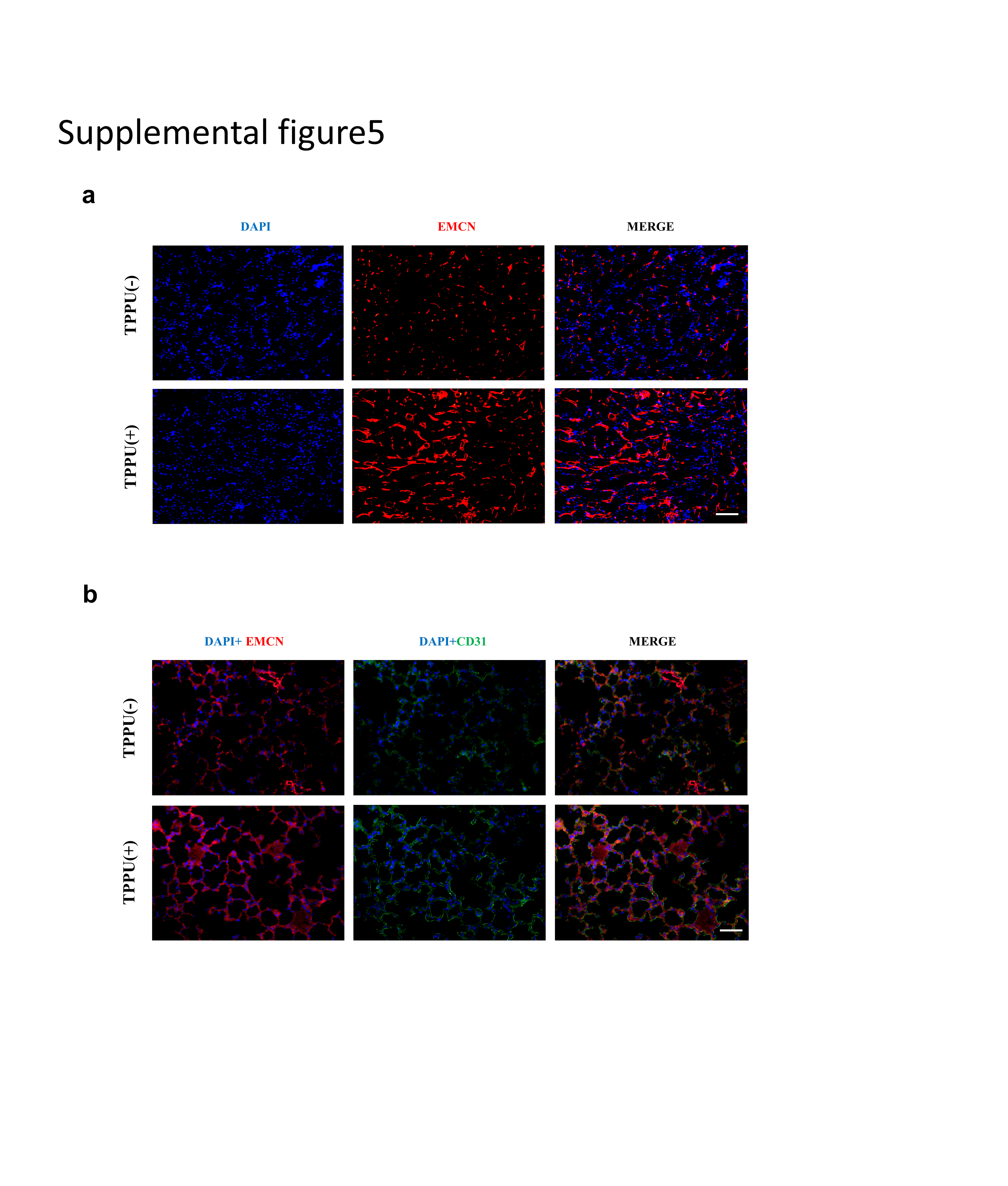

Supplement: Supplementary file 5 — FIGURE S5. TPPU treatment increases EMCN expression in lung and kidney [file CPR-56-e13403-s002.TIF]
