# Supplementary material for: Targeting soluble epoxide hydrolase promotes osteogenic–angiogenic coupling via activating SLIT3/HIF‐1α signalling pathway
Source: Cell Prolif. 2023 Jan 13;56(7):e13403. doi: 10.1111/cpr.13403 (PMC10334284; doi:10.1111/cpr.13403)
Supplement: Supplementary file 6 — TABLE S1. Primer sequence of the target genes TABLE S2. Target sequence of siRNA [file CPR-56-e13403-s003.docx]

**Supplemental Tables**

**Supplementary Table 1 Primer sequence of the target genes**

| Primers | Forward | Reverse |
| --- | --- | --- |
| GAPDH | GTGAAGGTCGGAGTCAACG | TGAGGTCAATGAAGGGGTC |
| CD31 | CCCCACTGAAGACGTCGAAT | TCTCCAGACTCCACCACCTTACTT |
| Emcn | TATTTTGCCGGTGGTTATTGC | AGAAGCTTCACGCTCTCTTTATCAG |
| Alp | ACCATTCCCACGTCTTCACATTT | AGACATTCTCTCGTTCACCGCC |
| Runx2 | AGCAAGGTTCAACGATCTGAGAT | TTTGTGAAGACGGTTATGGTCAA |
| Ocn | CTACCTGTATCAATGGCTGGG | GGATTGAGCTCACACACCT |
| Vegf | AGGAGTACCCTGATGAGATCGAGTA | TGGTGAGGTTTGATCCGCATA |
| Hif-1α | ACCGCTGAAACGCCAAAG | TCCATCGGAAGGACTAGGTGTCT |
| Slit3 | GAGAGGGAGCTTTCGATGGA | GTCATTACTCACACAGCCGATCA |
| Yap1 | GGGTGGCAATATTCAGTGCTTAAC | GTTATGATAGGCACACCCACAATTC |
| Robo1 | TCGCTGTTGGAGCAAACCATTA | CTGGCTGATGACTGAAGCAAGAA |

**Supplementary Table 2 Target sequence of siRNA**

| siRNA target genes | Target sequence |
| --- | --- |
| sh-Hif-1α | 5’- CCAGCAGACUCAAAUACAATT -3’ |
| sh-Slit3 | 5’- CGACUGAAUGACAAUGAGGUATT -3’ |
| sh-control | 5’- UUCUCCGAACGUGUCACGUTT -3’ |
